# Imbalance of laminar-specific excitatory and inhibitory circuits of the orbitofrontal cortex in autism

**DOI:** 10.1186/s13229-020-00390-x

**Published:** 2020-10-20

**Authors:** Xuefeng Liu, Julied Bautista, Edward Liu, Basilis Zikopoulos

**Affiliations:** 1 Human Systems Neuroscience Laboratory, Department of Health Sciences, Boston University, 635 Commonwealth Ave., Room 401D, Boston, MA 02215 USA; 2 Department of Anatomy and Neurobiology, Boston University School of Medicine, Boston, MA USA; 3 Graduate Program in Neuroscience, Boston University, Boston, MA USA

**Keywords:** Cortical layers, Myelinated axons, Inhibitory neurons, Calbindin, Parvalbumin, Calretinin, Prefrontal cortex, Amygdala, Emotions, Social interactions

## Abstract

**Background:**

The human orbitofrontal cortex (OFC) is involved in assessing the emotional significance of events and stimuli, emotion-based learning, allocation of attentional resources, and social cognition. Little is known about the structure, connectivity and excitatory/inhibitory circuit interactions underlying these diverse functions in human OFC, as well as how the circuit is disrupted in individuals with autism spectrum disorder (ASD).

**Methods:**

We used post-mortem brain tissue from neurotypical adults and individuals with ASD. We examined the morphology and distribution of myelinated axons across cortical layers in OFC, at the single axon level, as a proxy of excitatory pathways. In the same regions, we also examined the laminar distribution of all neurons and neurochemically- and functionally-distinct inhibitory neurons that express the calcium-binding proteins parvalbumin (PV), calbindin (CB), and calretinin (CR).

**Results:**

We found that the density of myelinated axons increased consistently towards layer 6, while the average axon diameter did not change significantly across layers in both groups. However, both the density and diameter of myelinated axons were significantly lower in the ASD group compared with the Control group. The distribution pattern and density of the three major types of inhibitory neurons was comparable between groups, but there was a significant reduction in the density of excitatory neurons across OFC layers in ASD.

**Limitations:**

This study is limited by the availability of human post-mortem tissue optimally processed for high-resolution microscopy and immunolabeling, especially from individuals with ASD.

**Conclusions:**

The balance between excitation and inhibition in OFC is at the core of its function, assessing and integrating emotional and social cues with internal states and external inputs. Our preliminary results provide evidence for laminar-specific changes in the ratio of excitation/inhibition in OFC of adults with ASD, with an overall weakening and likely disorganization of excitatory signals and a relative strengthening of local inhibition. These changes likely underlie pathology of major OFC communications with limbic or other cortices and the amygdala in individuals with ASD, and may provide the anatomic basis for disrupted transmission of signals for social interactions and emotions in autism.

## Background

Autism spectrum disorder (ASD) is a developmental disorder characterized by functional deficits in social behavior and emotional recognition [1–3]. There is mounting evidence highlighting the involvement of orbitofrontal cortices (OFC), among studies that have associated atypical organization of prefrontal cortical networks with the development of ASD pathology [4, 5]. The OFC is situated in the orbit, behind the eye socket, and has widespread connections with sensory association cortices, representing every sensory modality [6, 7], the anterior cingulate cortex (ACC) [8], and the amygdala [9–12], also reviewed in [13]. This connectivity pattern positions OFC as a key evaluator or integrator of sensory and emotional cues, and previous studies showed that OFC is implicated in emotion, social recognition, value updating, and decision-making [14–16].

Atypical OFC activity is observed in individuals with ASD [17, 18]. In line with this, OFC damage leads to symptoms seen in ASD, like rigid and obsessive compulsive behaviors, anxiety, and social apathy [19, 20]. In addition, presence of face cells in OFC may link atypical OFC activity with insensitivity to faces, as often seen in ASD [21]. Previous anatomical studies in ASD showed abnormality in OFC size and gross structure [22–26], as well as changes in axon morphology in the white matter below OFC [27–29]. At the cellular and neurochemical level, studies showed increased microglial activation [30] and atypical levels of neurotrophins and neurotransmitters [31–33]. These findings suggest changes in the overall growth and excitability of OFC networks, as well as excessive response of the immune system within OFC in ASD.

However, we know little about the underlying ASD pathology in the OFC gray matter, and its effects on OFC networks. To address this gap, we examined and compared the cortical structure of adult human OFC from neurotypical subjects and individuals with ASD at the cellular and single axon level. Our aim was to quantitatively describe the status of excitatory and inhibitory neurons and their axons that shape short- and long-range cortical communication and the balance of excitation/inhibition, which appear compromised in ASD.

## Methods

### Experimental design

**Fig. 1:**
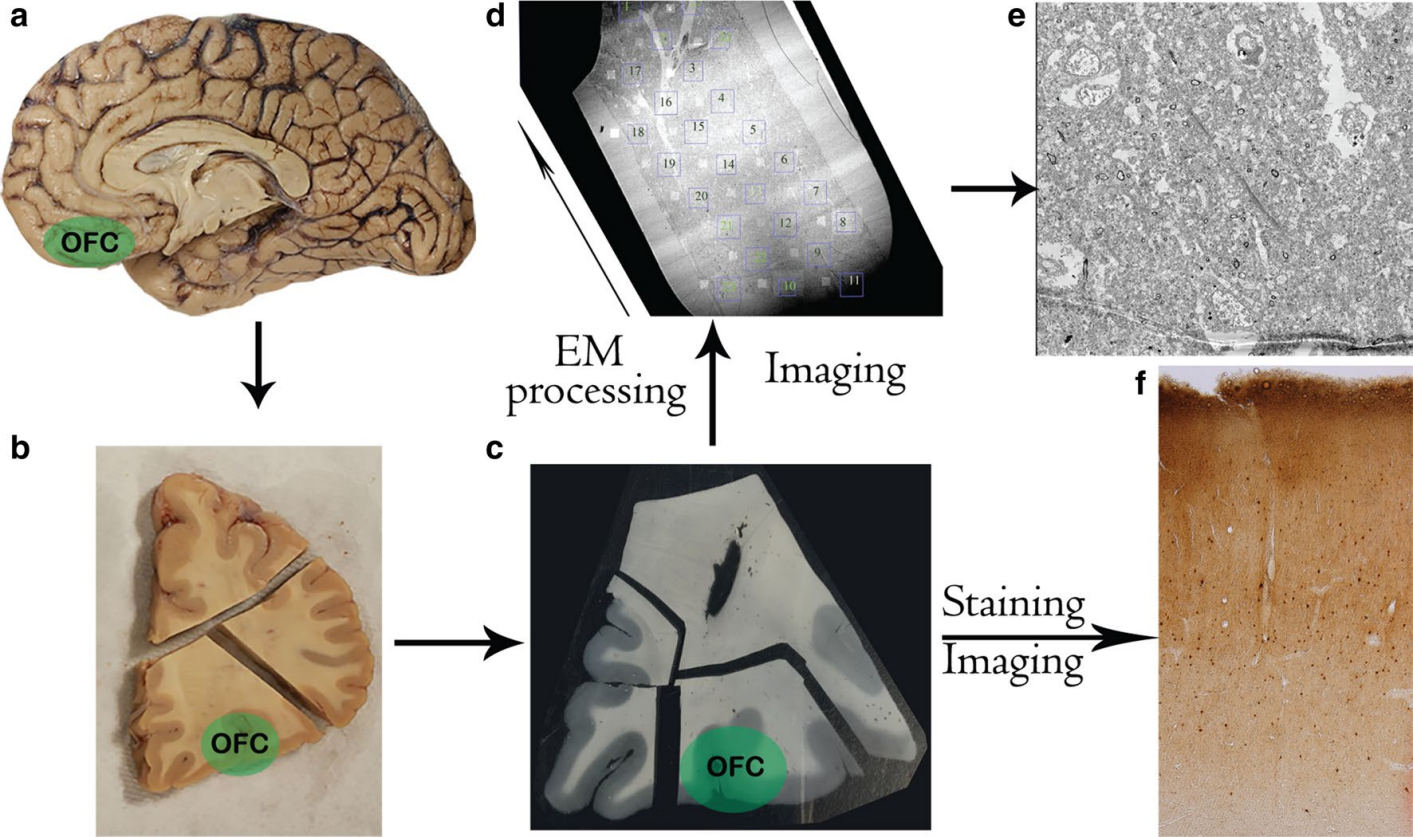
Experiment design. **a**, **b** Blocks of post-fixed post-mortem brain tissue containing OFC were separated (**b**) and cut into 50 μm thin sections in series (**c**). One batch of sections were processed for EM imaging. **d** A 100 nm ultrathin section was examined and multiple ROIs covering the entire section were imaged. **e** Example of high-resolution image from one ROI. **f** Adjacent sections were stained histologically to visualize all neurons or immunostained with antibodies against respective molecular markers to visualize inhibitory neurons (**f**)

The aim of this study was to examine the balance of excitation and inhibition in human OFC and its disruption in ASD. To do this we studied key excitatory and inhibitory components of OFC circuits, including the myeloarchitecture, cytoarchitecture and neurochemistry of OFC gray matter, in adults with or without autism. First, we examined the distribution and morphology of myelinated axon fibers across cortical layers, to paint a picture of excitatory networks in OFC. Then we examined the laminar distribution and morphology of three largely non-overlapping, neurochemically- and functionally-distinct types of inhibitory neurons in the local circuit, to assess the balance of excitation and inhibition in OFC circuits. Typical variability or irregularities in the laminar distribution and density of these excitatory and inhibitory circuit components likely underlies the functional integrity of layer-specific feedforward, feedback, cortical and subcortical OFC networks and their pathology in ASD. An overview of our experiment design and approach is shown in Fig. 1.

### Human post mortem brain tissue and sample size

Post-mortem brain tissue from 10 adults (6 Control, 4 ASD) was obtained from the Harvard Brain Tissue Resource Center through the Autism Tissue Program, Anatomy Gifts Registry, and the National Disease Research Interchange (NDRI). We used formalin-fixed tissue that was optimally prepared for correlated quantitative light and electron microscopy (EM) and immunohistochemical staining. To preserve the ultrastructure until processing, tissue blocks were cryoprotected in progressively increasing concentrations of buffered sucrose solutions (10–25% in 0.1 M PB) and were then immersed in antifreeze solution (30% ethylene glycol, 30% glycerol, 40% 0.05 M PB, pH: 7.4 with 0.05% azide) and stored at − 20 °C. Blocks of postmortem brain tissue containing areas of OFC were cut in 10 consecutive series of 50 μm sections. We then selected adjacent sections for EM processing and immunostaining experiments. We additionally fixed sections used for EM with 6% glutaraldehyde.

All available cases were included in qualitative and quantitative axon tracing and immunohistochemistry analysis. Our observations and previous published work [34] show consistent patterns of neuron and axon organization and density across layers in different cortices with comparable numbers of subjects. Each sample yielded a large number of data points, because we typically examine a large volume fraction of the areas sampled, and thus increase the number of individual axons and neurons examined. The high sampling fraction used in our studies minimized the variability within each analyzed case, and further increased statistical power, as we have described [34–38]. Our previous work [5, 27, 35–37] and power analysis showed that the sampling ratios exceed the samples needed to detect differences with a greater than 90% probability, and large effect population size (0.8) with ≤ 10% error, as recommended [39]. We used IBM SPSS Statistics 24 or PS version 3.1.6 (PS Power and Sample Size Calculations Program) for a posteriori power analysis of studies with an independent design that are analyzed by t-tests or regression. Input variables for power and sample size calculations included the Type I error probability (*α*), difference in population means (*δ*), regression errors or within group standard deviation (*σ*), slope of linear regression line (*λ*), and the ratio of control to experimental subjects (m). Data from these analyses are presented in the Results separately for overall OFC cell density counts using light microscopy, and EM studies. We further minimized variability, using only the OFC from right hemispheres of adults (mean age of Control group ± SD: 46.3 ± 12.9; mean age of ASD group ± SD: 36 ± 7.1) with similar PMI (mean PMI of Control group ± SD: 23.4 ± 5.4; mean PMI of ASD group ± SD: 25 ± 8.3). Clinical characteristics and other data on human subjects can be found in Table 1. The study was approved by the Institutional Review Board of Boston University (Protocol X3408). The diagnosis of autism was based on the Autism Diagnostic Interview-Revised (ADI-R) in all cases. Diagnosis information for ASD cases used can be found in Table 2.

**Table 1:** List of subjects and background information

Subject ID	Diagnosis	Sex	Hemisphere	Age	PMI (h)	Cause of death
176	Neurotypical (Control)	M	Right	67	30	Pancreatic cancer
2758	Neurotypical (Control)	F	Right	58	30	Pancreatic cancer
6004	Neurotypical (Control)	F	Right	36	18	Unknown
HCD	Neurotypical (Control)	M	Right	38	18.9	Cardiac Failure
HCU	Neurotypical (Control)	F	Right	39	20.1	Cardiac failure
HCO	Neurotypical (Control)	M	Right	40	23.7	Cardiac failure
Mean Control (± SD)				46.3 ± 12.9	23.4 ± 5.4	
4541	Autism	M	Right	44	31	Cardiac failure
5173	Autism	M	Right	30	20	GI bleeding
6232	Autism	F	Right	40	33	Respiratory arrest
6677	Autism	M	Right	30	16	Cardiac failure
Mean ASD (± SD)				36 ± 7.1	25 ± 8.3	

*PMI* post-mortem interval

**Table 2:** Further diagnosis of ASD cases

Subject ID	Score on autism diagnostic interview-revised			
	Social cutoff: 10	Communication cutoff (V): 8 cutoff (NV): 7	Restrictive and repetitive cutoff: 3	Early abnormal development cutoff: 1
4541^a^	26	18 (V), 13 (NV)	6	5
5173^b^	22	12 (NV)	2^a^	5
6232	12	14 (V)	8	5
6677	26	22 (V)	12	5

Other diagnosed disorders included, a: schizophrenia; b: seizures

*V* verbal communication score, *NV* non-verbal communication score

^a^Score was below cutoff threshold due to physical limitations and poor motor skills of donor. However, family members reported repetitive behaviors at a younger age. With this exception, which is not an unusual pattern in the behavioral domain, all donors had difficulties with communication, social behaviors, and atypical interests, consistent with a diagnosis of autism, and the ADI-R scores met and exceeded cutoffs for autism in each of these areas

### Immunostaining of inhibitory neurons and analysis

**Fig. 2:**
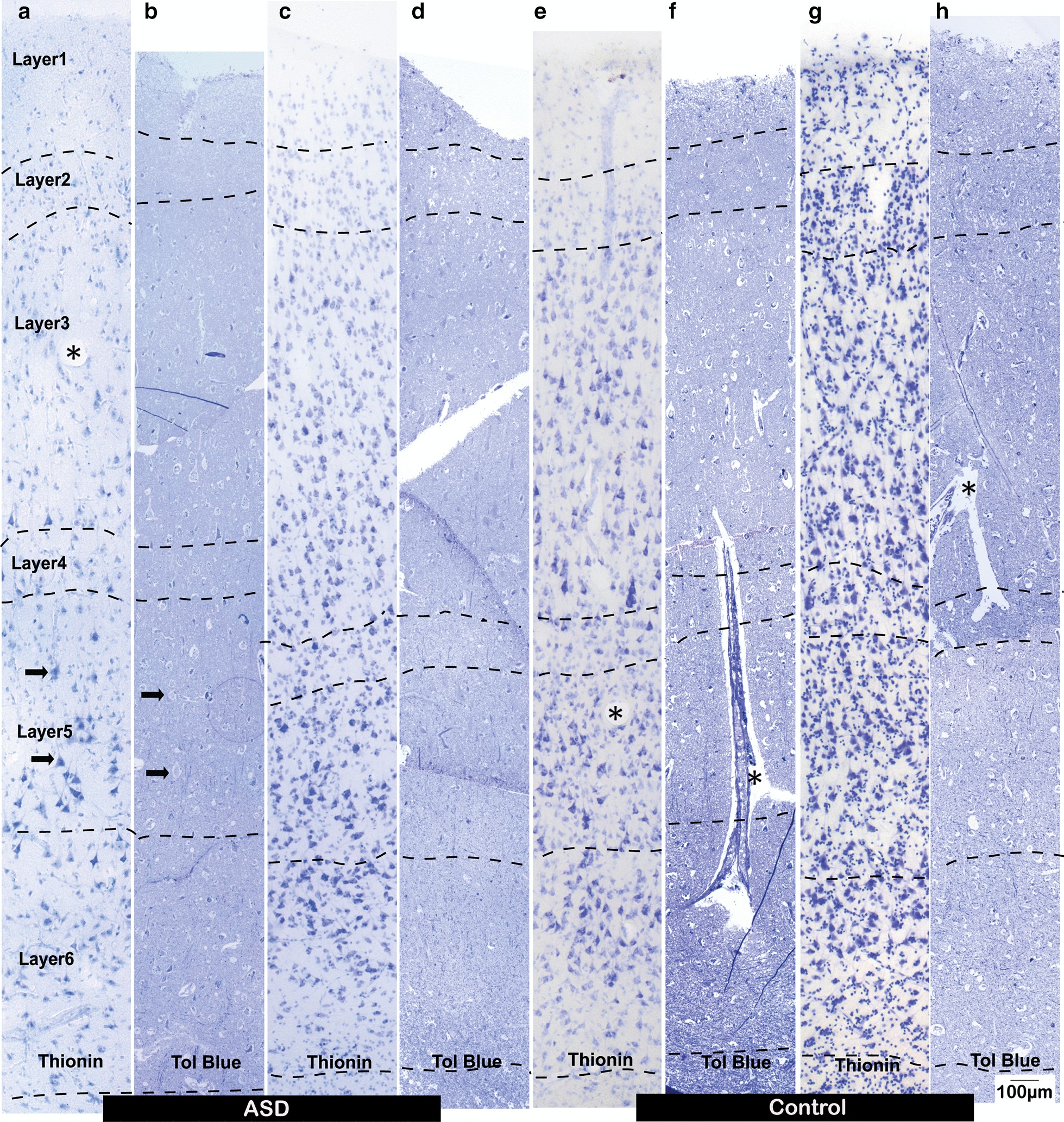
Cytoarchitecture of OFC column. Representative images of Nissl stained OFC sections from adults with ASD (**a–d**) and neurotypical adults (**e–h**). **a**, **c**, **e**, **g** Images of 50 µm sections stained against Thionin. **b**, **d**, **f**, **h** 1 µm thin sections from corresponding matching columns stained against Toluidine Blue, acquired from EM processed tissue blocks. Due to size limit of imaged tissue blocks, Toluidine Blue images are post-stitched. Arrows in **a** and **b** indicate examples of neurons. Asterisks “*” in **a**, **e**, **f** and **h** indicate examples of blood vessels

Histochemistry and immunostaining were performed as previously described [35, 37]. Briefly, we stained sections with Nissl (Thionin) to visualize the overall neuronal population in OFC (Fig. 2). Sections were pre-mounted on glass slides and air-dried before processing. Before staining, sections were defatted by incubation in chloroform/ethanol (1:1) mixture for 2 h, followed by gradual rehydration in graded alcohols. Then sections were stained in 0.05% of thionin blue for 15 min. Stained sections were dehydrated in increasing grades of alcohol solutions, cleared in Xylene, and coverslipped using Entellan (Merck).

To label the three types of cortical inhibitory interneurons, we used antibodies and immunostaining to identify their respective expression of calcium binding proteins, Parvalbumin (PV), Calbindin (CB), and Calretinin (CR). Briefly, adjacent sections were rinsed in 0.01 M PBS, pH7.4, and then incubated in blocking buffer (10% donkey normal serum, 5% of bovine serum albumin, and 0.1% of Triton X-100 in 0.01 M PBS) for 1 h, then incubated in respective mouse monoclonal or rabbit polyclonal primary antibody diluted in blocking buffer for 2 days at 4 °C (anti-PV: 1:2000, #P3088, Sigma; anti-CB: 1:2000, #CB-300, Swant; anti-CR: 1:2000, #6B3, Swant). Afterwards, the sections were rinsed in PBS and incubated for 3 h with donkey anti-mouse or donkey anti-rabbit biotinylated secondary antibodies (1:200, Vector Labs), then thoroughly rinsed in PBS. We then used avidin–biotin–peroxidase kit (Vector Labs) and diaminobenzidine (Zymed Laboratories) to visualize CB-, PV-, or CR-expressing neurons. After staining, sections were mounted and cover-slipped, following the same process as for Nissl staining.

We first examined Nissl stained sections to determine the contour and boundaries of each layer, using an Olympus BX-60 microscopy system equipped with Neurolucida and StereoInvestigator software (Microbrightfield) (Fig. 2). We used stereological principles and unbiased systematic sampling to count neurons in each layer and estimate their density. Specifically, we analyzed sections within series with a z-interval of 500 μm that was kept constant across cases. Two to three counting sites across the OFC area on a section were selected. To ensure consistency and minimize variability due to cortical folding that influences layer structure, cell morphology, and density [40], we analyzed counting sites from straight gyral segments and excluded segments in sulcal depths or near the top of a gyrus. Each counting site was a rectangular ROI covering the entire cortical column (pial surface to white matter) with average width of 500 µm. Overall, we sampled 9% of the OFC area in each section. In each counting site, we outlined 6 layers based on cellular morphology and density, further dividing each rectangular ROI into 6 laminar-specific counting sites. We counted neurons within each layer contour using the optical fractionator protocol (sampling grid size: 300 µm, counting frame (disector) size: 100 µm, disector thickness: 5 µm) under the microscope, using the 100X objective. Density of neurons was calculated using two approaches. First, we divided estimated neuron counts by estimated volume of each layer to get the packing density, which highlights the layers with the most densely packed neurons in OFC. We additionally calculated the relative density of neurons, by dividing estimated neuron counts by the estimated volume of the sampled ROI (all layers from pial surface to border with white matter). The relative density highlights layers with the highest number of neurons. To accurately and reliably identify neurons in Nissl stained sections, we used an established algorithm that facilitates distinction of neurons from glia and endothelial cells based on several key and uniquely-identifying features [38]. Briefly, these features include the intensity of nuclear staining (light for neurons, astrocytes, and endothelial cells and dark for oligodendrocytes and microglia), the presence of lightly stained cytoplasm for neurons, the shape and size of the nucleus, and the distribution of heterochromatin, which is different among distinct types of cells. We then applied and adjusted the same counting sites and contours to the adjacent immunostained sections, and using the 40X objective we counted inhibitory neurons in each outlined layer exhaustively, by setting the same grid and counting frame size (300 μm). We then calculated the relative and packing density of CB, PV, CR inhibitory neurons in each layer as previously. Numbers are presented as mean ± SE. We statistically analyzed estimates using ANOVA (Empowerstats) to compare Control and ASD groups. We also cross-validated the statistic comparison using a generalized linear regression (GLR) model, adjusting individual case heterogeneity and including PMI, age and sex as covariates. GLR analysis confirmed ANOVA results and did not reveal effects of PMI, age, and sex. Results from ANOVA analysis were presented in results and figures.

### EM processing

EM processing was done as previously described [35, 36]. Briefly, 50 µm OFC sections adjacent to matching Nissl- and immunostained sections were processed for EM using a high contrast method [35, 36]. Sections were first rinsed in 0.1 M PB and postfixed in 6% of glutaraldehyde. Then sections were rinsed in 0.1 M cacodylate buffer and then 0.1% tannic acid solution, followed by series of heavy metal solutions (1% osmium tetroxide with 1.5% potassium ferrocyanide, TCH aqueous solution (0.1 g of thiocarbohydrazide), and finally 2% osmium tetroxide) to induce heavy metal impregnation into lipid layers. We then washed the sections in distilled water and stained overnight in 1% uranyl acetate, followed by final stain in lead aspartate. Stained sections were then dehydrated in series of alcohols and cleared in propylene oxide. We then embedded sections in LX112 resin, sandwiched by thin sheets of Aclar film for long-term storage.

Before imaging, 1-mm wide rectangular segments containing desired OFC columns were cut out of the Aclar sandwich under a dissecting microscope, and each segment was then cut into 2 or 3 pieces (1X1 mm^2^), because the thickness of OFC is usually longer than the size limit of an EM imaging grid, and re-embedded in LX112 resin blocks for thin sectioning. Semi-thin sections (1 μm-thick) were cut and mounted on gelatin-coated slides and stained with Toluidine Blue Nissl solution, which stains all cells, the neuropil, and axons (Fig. 2). We used the stained sections to identify layer outlines and guide ROI selection for subsequent EM imaging. 100 nm-thick sections were cut and collected on single-slot pioloform grids for EM imaging.

### EM imaging and analysis

**Fig. 3:**
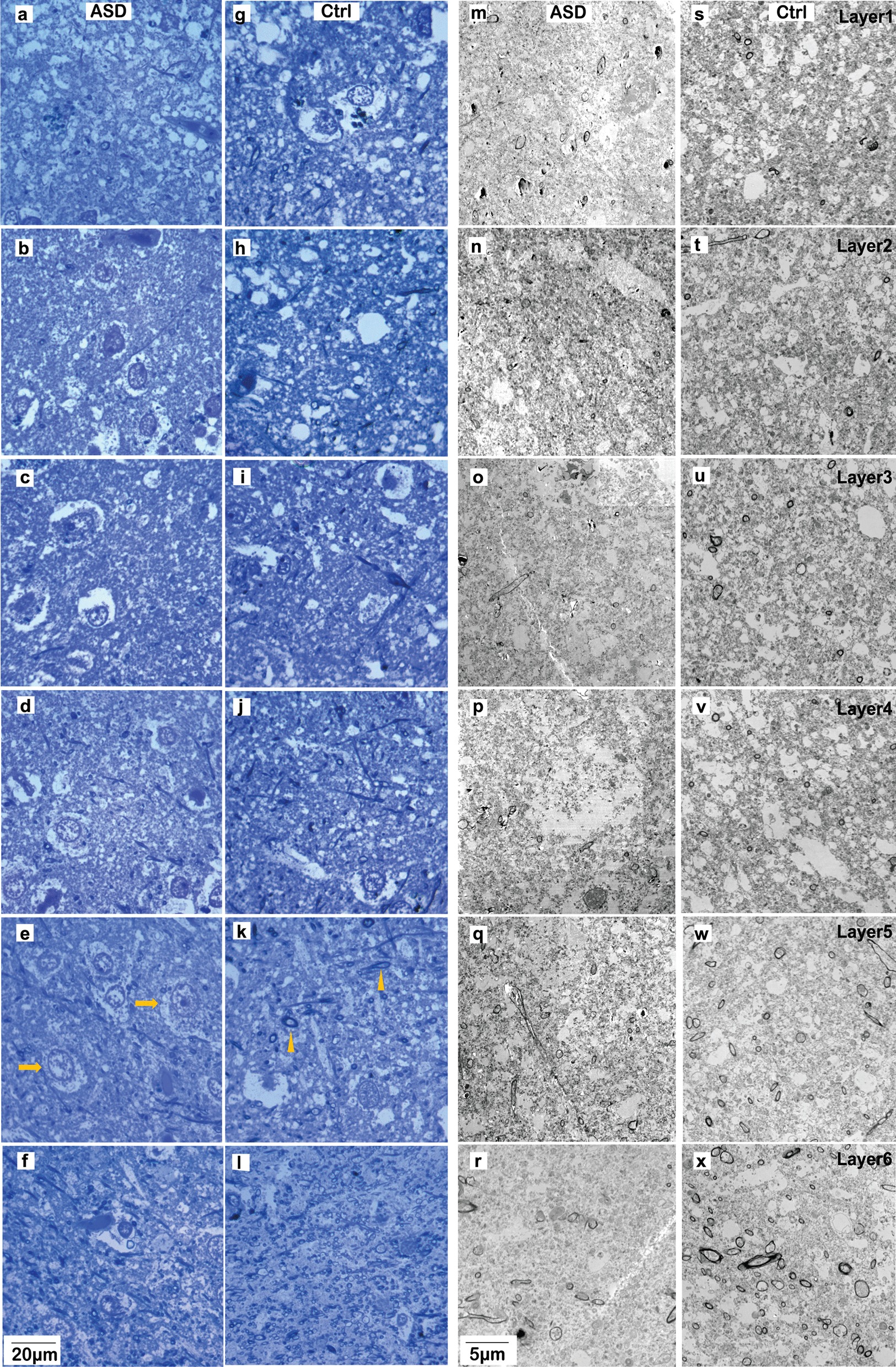
Laminar distribution of myelinated axons in the OFC of adults. Examples of Toluidine Blue staining viewed under the light microscope (**a**–**l**) and EM images (**m**–**w**) acquired from OFC sections. **a**–**f**, **m**–**r** Representative images from OFC layers 1–6 of adults with ASD. **g**–**l**, **r**–**w** Representative images from OFC layers 1–6 of neurotypical adults. Cross-sections of myelinated axons appear as ring or elongated oval shaped structures. The morphometrics and density of axon cross-sections were quantified and compared between ASD and Control groups. Arrows in **e** indicate examples of neuron somata. Arrowheads in **k** indicate examples of myelinated axon cross-sections

We acquired high-resolution (30 nm/pixel) images using a scanning electron microscope (Zeiss Gemini 300 with STEM detector, Atlas 5 software). We used tissue from 4 Control and 4 ASD subjects and imaged 268 square ROIs (120 × 120 μm), systematically sampling the entire section, with sampling ratio over 15%. In total, we analyzed 169 ROIs in the Control group and 99 ROIs in the ASD group, averaging 45 ROIs per layer (range from 19 to 74, depending on the size of the layer). Using matching Toluidine Blue stained sections as reference, we grouped images by layers for morphometric analysis (Figs. 2, 3).

We analyzed EM images with ImageJ (NIH). Cross-sections of myelinated axons in each image were identified, morphometrics were estimated, and summarized into a list. Measured morphometrics were grouped for comparison, and included axon size, profile area, and shape indicators (min and max diameters and circularity) that were used to estimate axon density (number of cross-sections per unit), surface area ratio (total surface area/image area), and minor diameter (excluding myelin) as indication of thickness. In addition, axon trajectory was estimated by the angle of the cross-section, as described previously [36]. We estimated the variation of angle values in each sampled image, as a measurement of axon trajectory variability. Numbers are presented as mean ± SE. We used ANOVA to compare morphometrics between Control and ASD groups (Empowerstats). We also cross-validated the statistic comparison using a generalized linear regression (GLR) model, adjusting individual case heterogeneity and including PMI, age and sex as covariates. GLR analysis did not reveal effects of PMI, age, and sex and confirmed ANOVA analysis. Findings from ANOVA analysis were presented in results text and figures. In addition, we also used a repeated measures ANOVA followed by Tukey’s post-hoc tests to further statistically assess and confirm the effects of ASD on the myelinated axon population within the OFC gray matter. Because EM is a high-resolution, but low throughput approach, with relatively low N of subjects, a repeated measures design can also be typically used, wherein different sections, blocks of tissue, or regions of interest from each case can be pooled and compared. This framework formed the basis for a posteriori power analysis, performed as described above, to estimate appropriate sample size and power for the EM studies. All statistical analysis approaches used for the EM studies provided similar results, highlighting the same significant changes between Control and ASD groups.

## Results

### Altered axon morphometrics in OFC layers

**Fig. 4:**
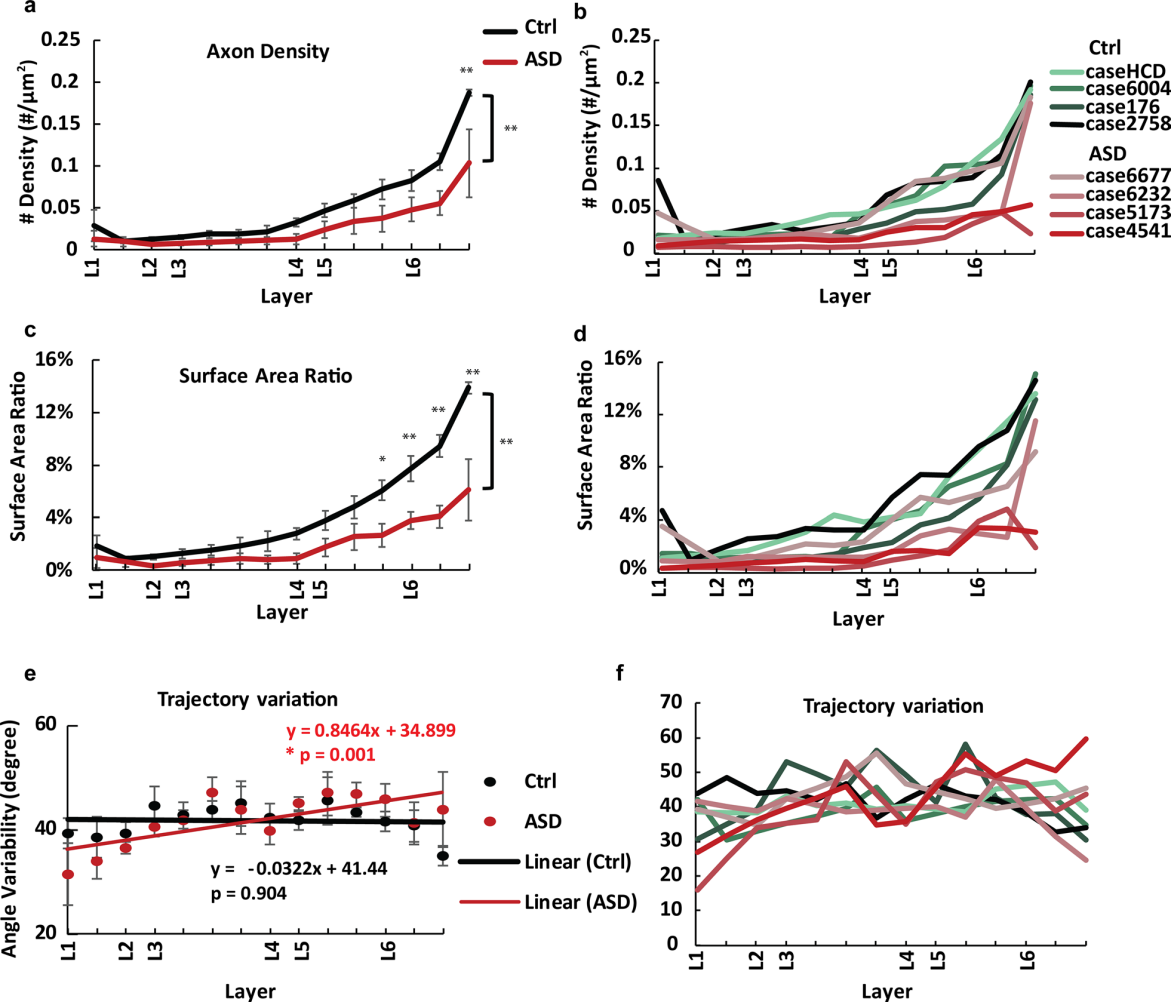
Myelinated axons in OFC showed different density and trajectory variability in adults with ASD compared to neurotypical controls. **a** Mean density (± SE) of myelinated axons in ASD OFC was significantly lower than controls across the column, both by axon number density (**a**) and by total surface area ratio (**c**). The difference was most pronounced in deep layers. Bracket and asterisks (**) indicate significant difference between two groups found by ANOVA (*p < 0.05; **p < 0.01), while asterisk on top indicates significant difference of that layer location found by post-hoc analysis. Layer marks on the horizontal axes indicate the relative starting position of each layer across the column. The density maps of individual cases are presented in (**b**, **d**) respectively. **e** Distribution of mean axon trajectory variation followed different trends between ASD and Control groups. While the linear regression in the Control group showed a generally constant (flat) trend across the column (no difference of slope from 0, *p* = 0.904), the linear regression in the ASD group showed increasing trajectory variability towards deeper layers (significant difference of slope from 0, *p = 0.001). **f** Axon trajectory variation across the column for each individual

**Fig. 5:**
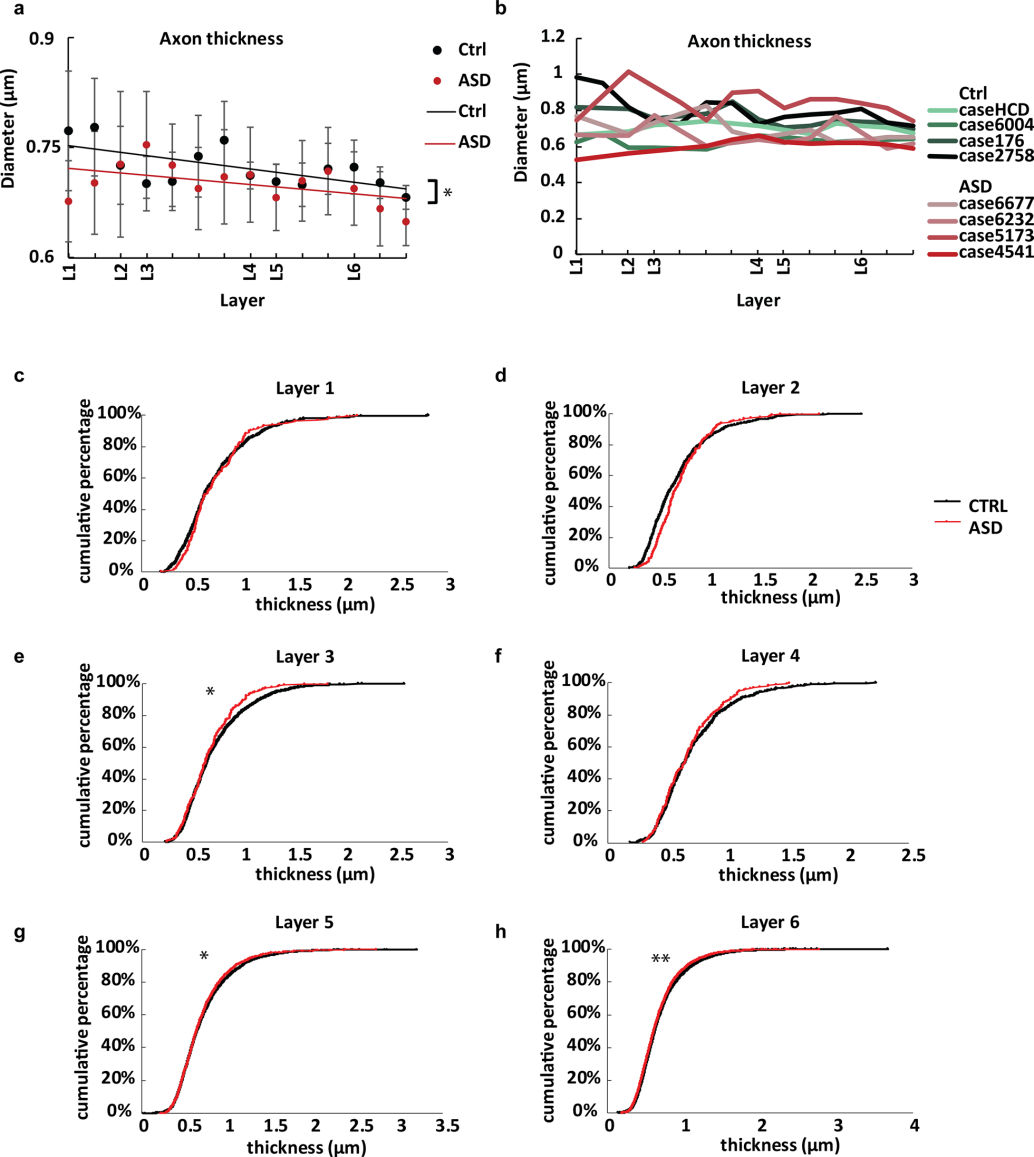
Myelinated axons in OFC showed different morphometrics in adults with ASD compared to neurotypical controls. **a** Axon thickness, measured as minor diameter of axon cross-section, was significantly smaller in ASD compared with the Control group across all layers (ANOVA, *p* = 0.039). **b** Average axon thickness data for each individual across layers. **c–h** Cumulative plots of axon thickness data. Each line represents pooled thickness data of the group from the respective layer. ASD curves showed a general shift towards thinner axons, especially in deep layers (**e**–**h**). In layers 1 and 2, the ASD group curves showed a shift towards thinning only of thick axons, and a slight thickening of thinner axons, compared to controls. * indicates significant difference (*p* < 0.05, *t* test) on average thickness of pooled axons from that layer between two groups, ** indicates *p* < 0.01

In order to examine how neural transmission, especially excitatory transmission, is organized in OFC, we analyzed the distribution and morphology of myelinated axons across the entire cortical column in a systematic manner (Figs. 2, 3, 4, 5). Power analysis based on pooling of ROIs from 4 experimental (99 ROIs) and 4 control subjects (169 ROIs; repeated measures design) was used to estimate sample size and power for axon size and density estimates. Overall, our measurements yielded normally distributed estimates within each subject group with an estimated standard deviation and an average difference in the ASD and control means that allowed us to reject the null hypothesis that the population means of ASD and Control groups are equal with probability (power) 0.9. The Type I error probability associated with the test of this null hypothesis was 0.05.

Myelinated axons are predominantly from excitatory projection neurons, most of which form short- and long-range incoming and outgoing projections. In line with previous findings [37], we found that the density of myelinated axons in neurotypical OFC increased consistently from layer 2 through 6 however, axon density in layer 1 appeared higher than in the neighboring layer 2 (Fig. 4a–d). Inner axon diameter did not change significantly across layers (Fig. 5a, b. n = 4; *p* = 0.371 ANOVA). In OFC of individuals with ASD, we found that the distribution of myelinated axons followed a similar trend as in the neurotypical group. However, the density of myelinated axons in ASD was significantly lower than in controls across all layers, both by number (Fig. 4a, b; n = 4 for ASD, n = 4 for control; *p* < 0.001, ANOVA) and by total surface area (Fig. 4c, d; *p* < 0.001, ANOVA). The difference was more pronounced in the deep layers. Post-hoc analysis showed significant difference on axon number density in layer 6 (*p* < 0.01), and significant difference on surface area density in layers 5 and 6 (*p* = 0.029 for layer 5, and *p* < 0.01 for layer 6). These were the layers with the highest density of myelinated axons and relatively low variability within each group. The trends suggest a general reduction of excitatory innervation in OFC in individuals with ASD.

We then analyzed the trajectories of axon profiles. Variation of axon orientation of neurotypical OFC showed a flat trend from top layers to deep layers, while axons in ASD OFC showed increasing orientation variation towards deeper layers, suggesting disorientation or more branching of axons (Fig. 4e, f). We also compared the inner diameter of myelinated axons between ASD and Control groups. Average axon diameter differed significantly between the two groups with an overall higher proportion of thin axons in ASD across all layers (Fig. 5a, b; *p* = 0.039, ANOVA). The thinning of axons in ASD was especially pronounced in deep layers, whereas towards superficial layers 1 and 2 diameters of myelinated axons showed a shift towards medium thickness, compared with the Control group. Average thickness of pooled axons from control group was significantly lower than that in the ASD group, at layers 3, 5 and 6 (t-test; Layer 3: *p* = 0.021; Layer 5: *p* = 0.035; Layer 6: *p* < 0.01; Fig. 5c–f). Again, these were the layers with the highest density of myelinated axons and relatively low variability within each group.

Estimates of axon size showed relatively higher variability, especially within the ASD group, possibly reflecting heterogeneity of ASD symptoms and comorbidity with other disorders. In particular, case 5173 (diagnosis of ASD and epilepsy) had thicker OFC axons on average, in line with previous studies that correlated enlarged axon diameter with epilepsy [41]. We could not reliably assess the effects of comorbid diagnoses in our limited sample however, we were able to reliably detect significant differences despite the observed variability. Excluding 5173 from analyses, further amplified the differences in axon thickness between the two groups (mean difference Control vs ASD = 0.072 µm; *p* < 0.01 ANOVA, not shown), compared with results including all cases that still showed significant differences (mean difference Control vs ASD = 0.020 µm; *p* = 0.039, Fig. 5a).

Finally, we also separated all axons within each respective group (Control and ASD) into 3 categories based on thickness (thin: diameter < 0.8 µm; medium: 0.8–1.5 µm; thick: diameter > 1.5 µm), and estimated the ratio of thin, medium, and thick axons in all layers, as described before [27, 35–37]. This analysis showed that the ASD group had significantly more thin axons compared to the Control group (Control: 69.2% ± 2.9% vs ASD: 78.7% ± 3.3%; *p* < 0.01, ANOVA), but fewer medium and thick axons (medium, control: 27.5% ± 2.2% vs ASD: 20.1% ± 2.7%; *p* < 0.01, ANOVA; large, control: 3.3% ± 1.1% vs ASD 1.2% ± 0.7%; *p* < 0.01, ANOVA) in all layers.

### Inhibitory neuron network in OFC layers

**Fig. 6:**
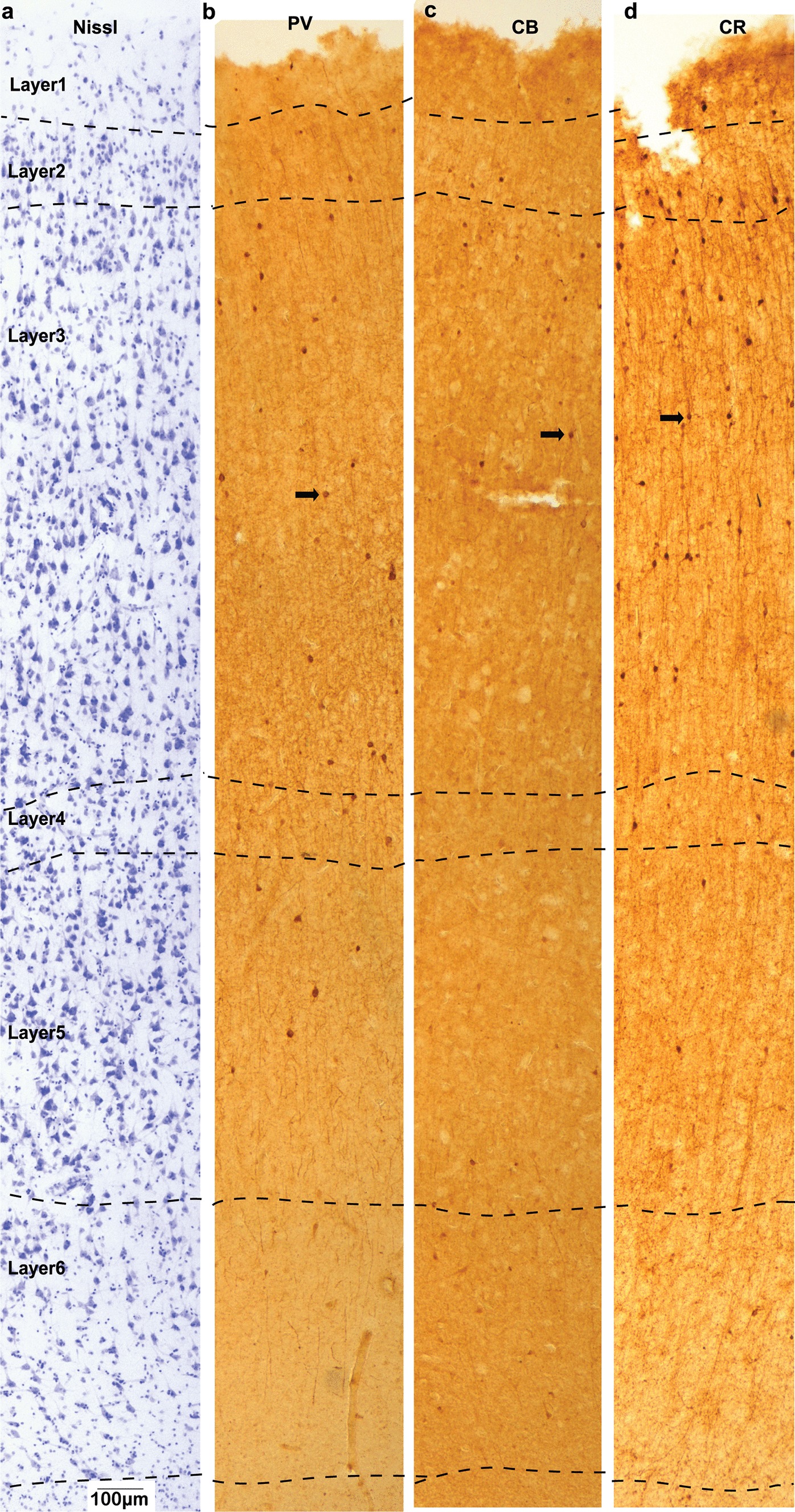
Representative images of OFC column from neurotypical adults. Adjacent sections were Nissl stained to visualize general cytoarchitecture of OFC column (**a**), as well as against Parvalbumin (**b**), Calbindin (**c**), and Calretinin (**d**) to visualize distribution of three major types of inhibitory neurons. Arrows in **b**, **c** and **d** indicate examples of stained neurons

**Fig. 7:**
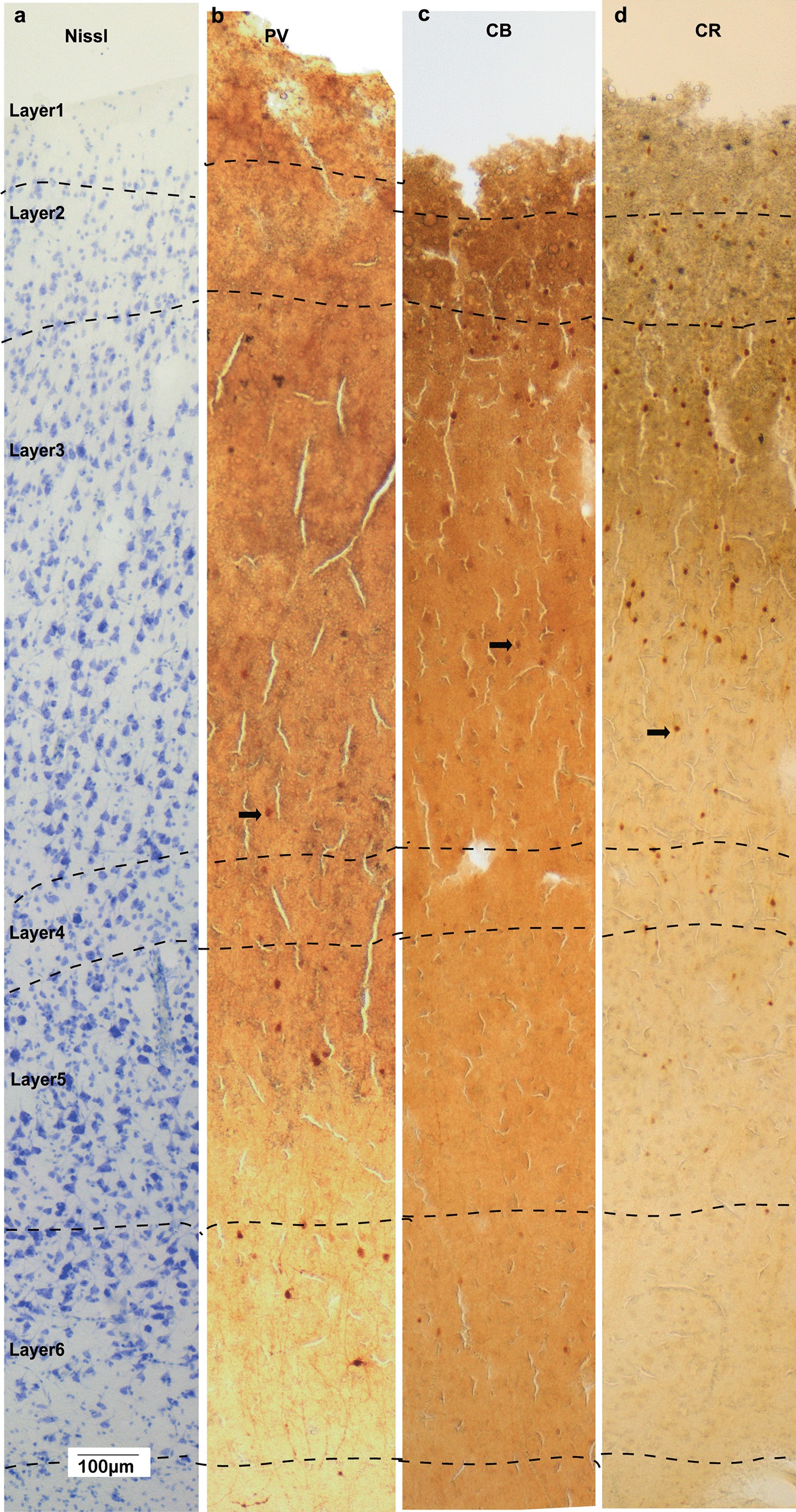
Representative images of OFC column from adults with autism. Adjacent sections were Nissl stained to visualize general cytoarchitecture of OFC column (**a**), as well as against Parvalbumin (**b**), Calbindin (**c**), and Calretinin (**d**) to visualize distribution of three major types of inhibitory neurons. Arrows in **b**, **c** and **d** indicate examples of stained neurons

**Fig. 8:**
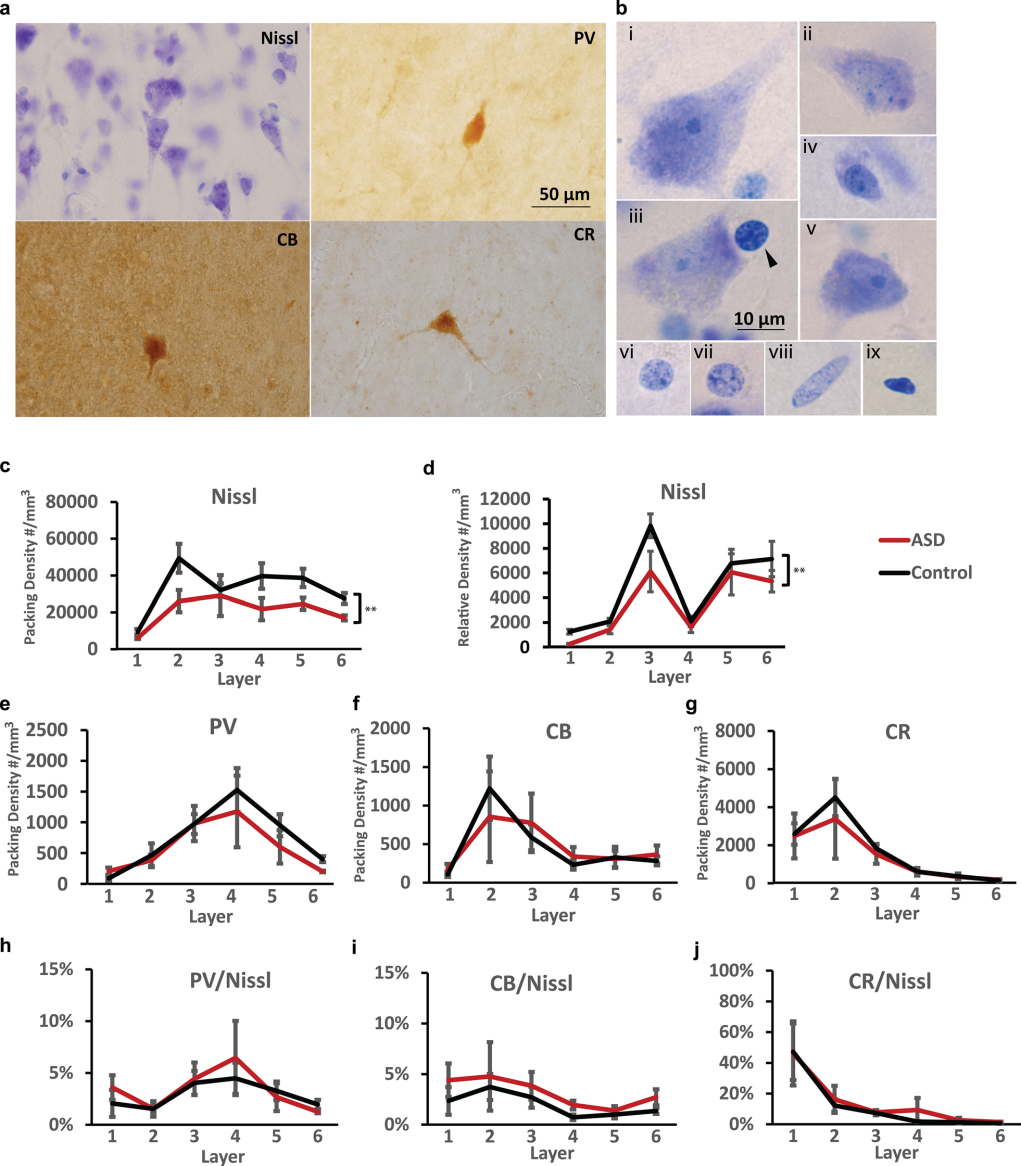
Overall reduction in OFC neuron density in adults with ASD. (**a**) Examples of OFC coronal sections stained with Nissl or antibodies against the three main markers of inhibitory neurons (PV, CB, and CR) in OFC. (**b**) Example of different types of cells in OFC visualized by Nissl stain: (i–v) examples of neurons. Note example of an oligodendrocyte in (iii) near a neuron (arrowhead). (vi, vii) examples of astrocytes. (viii) Example of an endothelial cell. (ix) Example of microglia. **c, e, f, g** Packing density of different cell populations, estimated as number of cells in a layer per volume of respective layer. (**d**) Relative density of total neurons, estimated as number of Nissl stained neurons in a layer per volume of entire included column. **c**, **d** Total neuron density in the ASD group was lower than the Control group. **e**–**g** The densities of three major types of inhibitory neurons, PV, CB and CR, were not significantly different between groups. **h**–**j** The density of all three types of inhibitory neurons relative to total neuron population showed no significant differences between groups

We examined the expression pattern of PV, CB, and CR inhibitory neurons, using immunolabeling. In addition, we stained the entire neuron population with Nissl to determine the laminar ratio of excitatory and inhibitory neurons in OFC (Figs. 6, 7, 8). Examples of different types of cells stained with Nissl are shown at high magnification in Fig. 8b, and appeared similar to cell types reported in previous studies of the primate cortex [37, 38, 42]. Neurons were characterized by visible cytoplasm around the nucleus and presence of a dark nucleolus in the center of the lightly labeled nucleus (Fig. 8b i–v). Astrocytes were characterized by a thick rim of peripheral heterochromatin, and unstained cytoplasm (Fig. 8b vi, vii). Other types of cells included oligodendrocytes (darkly-stained nuclei with round or oval shape, often present in the vicinity of large neurons as satellite glia, Fig. 8b iii), endothelial cells (lightly-stained nuclei, often elongated and surrounding blood vessels, Fig. 8b viii), as well as microglia (darkly-stained nuclei with irregular shape, Fig. 8b ix).

The overall density of neurons in all OFC layers (Mean ± SE) in the Control group was 29,165 ± 3050 neurons/mm^3^ (Fig. 6), in line with previous estimates in similar dysgranular medial and ventromedial prefrontal cortices and slightly lower than relatively more eulaminate anterior OFC regions (areas 11 and anterior 13), as we have reported previously [37]. The overall density of neurons in all OFC layers in the ASD group was significantly lower 18,845 ± 2523 neurons/mm^3^ (Figs. 7, 8; ANOVA p = 0.028, F *crit* = 5.32). Specifically, for overall OFC cell density counts using light microscopy, our design with 4 experimental subjects and 6 control subjects yielded normally distributed estimates within each subject group with standard deviation that averaged 4370 cells/mm^3^ and an average difference in the ASD and control means of 10,319 cells/mm^3^. Based on these estimates we were able to reject the null hypothesis that the population means of ASD and Control groups are equal with probability (power) 0.9. The Type I error probability associated with the test of this null hypothesis was 0.05.

In neurotypical OFC, Nissl staining showed that layers 2 and 4 have the highest density of neurons among layers, in line with previous studies of primate OFC (Fig. 8c) [37, 43]. However, layers 3, 5, and 6, which are the largest layers with most projection neurons, have the highest number of neurons within the OFC cortical gray matter column (Fig. 8d). The highest density of CR-positive neurons was found in layers 1 and 2. The density of CB-positive neurons was highest in layers 2 and upper layer 3, while PV-positive inhibitory neurons were mostly found in the middle/deep layers.

In ASD, the general distribution pattern of neurons remained the same (Fig. 8). However, neuron density was partially altered. At the laminar level, we found that the density of the overall neuron population appeared significantly lower in the ASD group across all layers, both estimated by packing density (Fig. 8c; Nissl: *p* < 0.001; ANOVA) or relative density (Fig. 8d; Nissl: *p* < 0.006; ANOVA). The general distribution pattern and density of the three types of inhibitory neurons in ASD were not significantly different from neurotypical controls (Fig. 8e–g; n = 6 for control, n = 4 for ASD; PV: *p* = 0.065, CB: *p* = 0.915, CR: *p* = 0.467; ANOVA), although densities of PV and CR neurons showed slight trends of reduction in ASD group. Overall, neurons in the ASD group showed a trend for higher relative ratio of inhibitory/total neurons compared with the control group however, no significant difference was found (Fig. 8h–j; PV: *p* = 0.528, CB: *p* = 0.053, CR: *p* = 0.391; ANOVA).

## Discussion

In this study, we examined the balance of excitation and inhibition in human OFC and its disruption in ASD. Our study focused on key excitatory and inhibitory components of OFC circuits, including the myeloarchitecture, cytoarchitecture and neurochemistry of OFC gray matter, in adults with or without autism. We described the quantitative distribution, trajectory, and morphology of myelinated axon fibers and the density of excitatory neurons across cortical layers, as a proxy of excitatory networks in OFC. We then examined the laminar distribution and morphology of three largely non-overlapping, neurochemically- and functionally-distinct types of inhibitory neurons in the local circuit. These three types of inhibitory neurons are characterized by expression of specific calcium-binding proteins PV, CB and CR, and constitute the major source of cortical inhibition [44]. Using cellular and single-axon high-resolution quantitative approaches, we established, for the first time in our knowledge, the typical laminar distribution, density, and relationship of excitatory and inhibitory circuit components in the human OFC. Finally, we identified atypical changes that likely underlie layer-specific feedforward, feedback, cortical and subcortical OFC network pathology and imbalance of excitation and inhibition in OFC circuits in ASD.

Our study of the neurotypical human OFC gray matter at the single axon and cell resolution across layers complements and extends previous research on the trajectory and morphometrics of axon bundles in OFC white matter with diffusion tensor imaging (DTI) and high-resolution neuroanatomical approaches [27, 45]. We found that myelinated axon density in neurotypical OFC decreased from layer 1 to layer 2 and then increased gradually towards the deeper layers that are closer to the white matter, following a consistent pattern across human and non-human primates [37, 43, 46]. Axon thickness remained stable across layers. Compared with previous reported results in the white matter below OFC [27], myelinated axons in the gray matter were thinner, indicating overall thinning of axons after entering into the gray matter, but no further tapering within the gray matter. Myelinated axon profile orientation in our sections was used as a proxy of axon trajectory in 3D. Analysis showed that axon orientation variability appeared to be consistent across layers in OFC gray matter, but more heterogeneous than previously reported in the superficial white matter below prefrontal cortices [36]. Taken together with previous studies, our findings show that axons become thinner and their trajectory becomes more heterogeneous, likely due to increased branching, as they transition from deep to superficial white matter, and finally enter the gray matter [27, 36].

Excitatory and inhibitory neuron distribution and density in the neurotypical OFC were in line with previous reports in primate OFC and other cytoarchitectonically similar prefrontal cortices [37, 47–49]. Narrow cortical layers 2 and 4 that had the highest density of neurons showed relatively low density of myelinated axons, whereas thicker layers with lower neuronal density had more room for myelinated axons. Inhibitory neurons accounted for about 20% of all neurons in OFC, in line with previous studies in the frontal cortex of humans [50–54]. In primates, inhibitory neurons can be classified by label with the calcium binding proteins PV, CB or CR, which comprise largely non-overlapping neurochemical groups of cortical inhibitory neurons [44, 55, 56]. PV labels basket and chandelier inhibitory neurons [57, 58], which are most prevalent in the middle cortical layers, where they form perisomatic synapses and strongly inhibit pyramidal neurons. CB labels several cortical morphologic types of inhibitory neurons, found most densely in cortical layers 2 and 3, and innervate distal dendrites of pyramidal neurons, modulating their activity [59]. CR inhibitory neurons are also found mostly in the upper layers (1–3a), where they innervate mostly other GABAergic neurons [60–62]. This regularity in the laminar distribution of PV, CB, and CR neurons has been shown in frontal, temporal and sensory association areas, which have been studied in primates [63–67], and was evident in the OFC of neurotypical adults examined here.

In the OFC of adults with ASD, the balance of excitatory and inhibitory network components was disrupted. We found that, in general, myelinated axons were less dense, and thinner in the ASD group, suggesting weaker excitatory transmission in OFC networks in ASD. Importantly, estimates of axon size showed relatively higher variability, especially within the ASD group, possibly reflecting heterogeneity of ASD symptoms and comorbidity with other disorders. Despite the heterogeneity of ASD however, axon changes were highly and consistently significant. Lower density of myelinated axons, together with observed axonal thinning and heterogeneity of axon trajectory, as indicators of branching [35, 36], may also indicate increased relative presence of unmyelinated axons and upregulated local circuit communication.

**Fig. 9:**
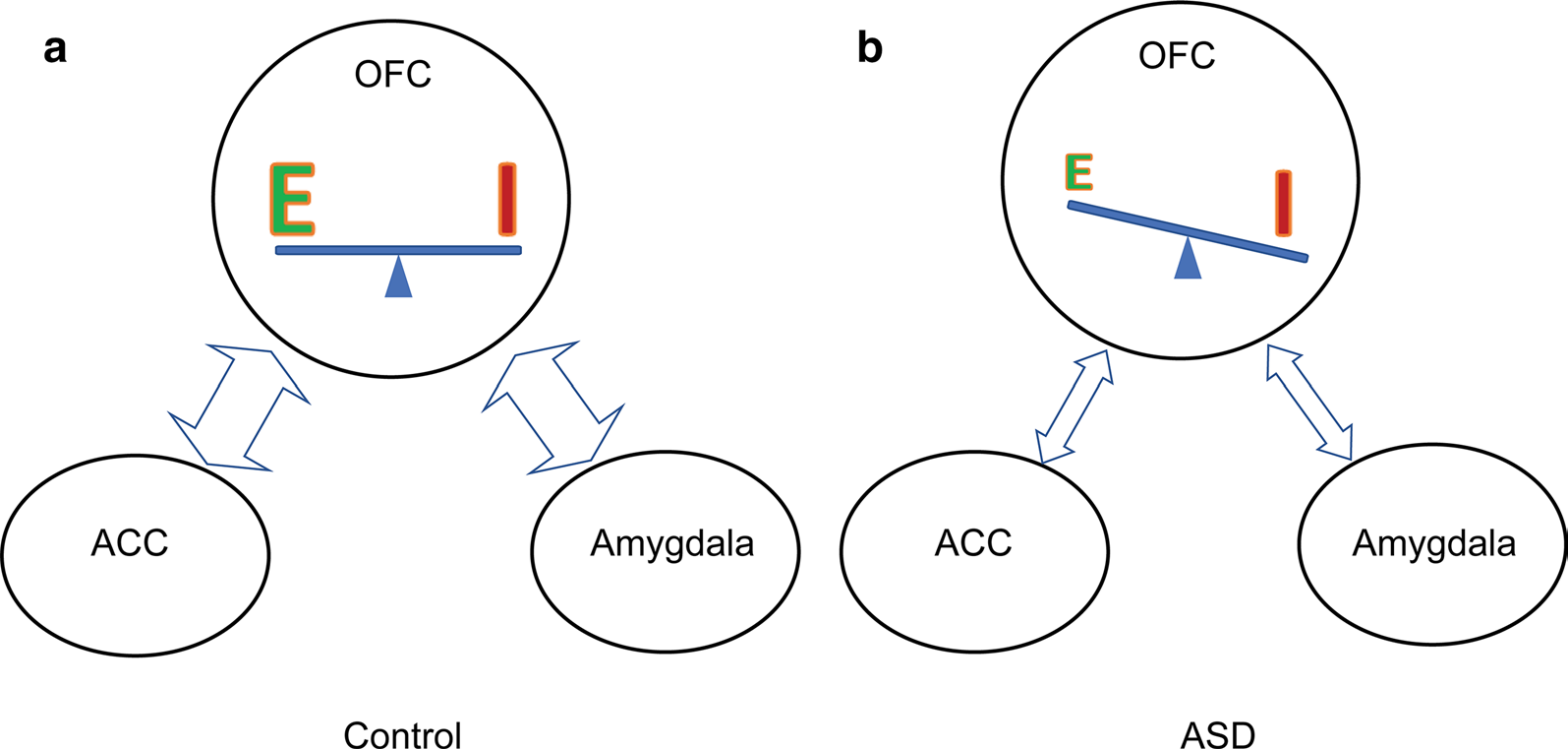
A schematic diagram of a model summarizing major OFC connectivity networks in neurotypical adults (**a**) and their likely disruption in ASD (**b**). Our findings suggest that the balance of excitation/inhibition (E/I) is disrupted in OFC, with an overall weakening of excitatory inputs and outputs, tipping the scales due to the relative increase in the local inhibitory load (strengthening of inhibition) in all cortical layers of OFC. These changes likely affect robust short-range reciprocal connections with cytoarchitectonically similar limbic medial prefrontal cortices (MPFC) and anterior cingulate cortices (ACC), which involve most cortical layers (lateral or columnar connectivity pattern, gray arrows). Feedforward (purple arrows) and feedback (blue arrows) long-range pathways linking OFC with sensory association areas, mostly in the temporal lobe, and the amygdala are also likely disrupted in ASD. Arrows indicate reciprocal connections and their thickness indicates relative strength

Neuron analysis in each layer showed that the overall neuron population was lower in OFC of adults with ASD, while the density of inhibitory neurons did not change significantly compared to the controls. Therefore, our findings indicate that the reduction in neuron density mainly involved excitatory neurons. Combined with the density reduction and thinning of myelinated axons in OFC, which are predominantly excitatory, our data support a universal weakening of input and output, or downregulation of activation levels in OFC of adults with ASD (Fig. 9). However, given the heterogeneity of ASD, limited sample size, and inherent variability of estimated features, more studies will be necessary to address changes in inhibition in OFC of individuals with ASD. Nevertheless, our findings provide anatomical evidence, supporting previous functional imaging studies, which showed reduced OFC activation and information flow between OFC and neighboring areas in individuals with ASD [20, 68].

The laminar-specific disruption of excitatory cortical neurons and myelinated axons, and the resulting imbalance in excitation/inhibition can provide further insights about network pathology in ASD, including the integrity of short- and long-range feedforward and feedback OFC connections (Fig. 9). ASD is characterized by disruption in sensory processing, attention, social interaction and value-based decision-making. OFC connections with sensory association cortices, limbic medial prefrontal cortices, including the ACC, and the amygdala, position OFC as a key node for the integration and evaluation of sensory information and emotional states [6, 8, 10–13, 69].

In particular, the posterior OFC receives input from unimodal and multimodal association cortices from all sensory modalities (reviewed in [47]). The sensory input helps OFC calibrate information in a timely manner. In primates, the connections originate mostly from the upper layers of sensory association cortices and innervate mainly the middle OFC layers (3–5), in a feedforward manner [70, 71]. In turn, the OFC can also modulate sensory processes, especially auditory processing [72, 73], through feedback connections. Impairment in sensory processing and evaluation, often associated with ASD [74, 75], involves disengagement of sensory cortices and neural circuits that process social reward, in which OFC has a central role [76]. In addition, previous studies have linked under-connectivity between auditory processing circuits and OFC with insensitivity to human voice in individuals with ASD [18]. Our findings, suggesting weakening of excitatory inputs and outputs in OFC and a relative strengthening of local inhibition, provide the anatomical substrate for the disruption of these long-range connections in ASD. However, it is worth noting that some individuals with ASD accompanied by sensory hypersensitivity show heightened OFC activity [17], therefore more studies are needed to clarify the role of OFC networks in sensory processing.

The OFC is also robustly interconnected, through short/medium-range pathways, with medial prefrontal cortices, including neighboring ACC and ventromedial subgenual area 25, in primates [13, 77–79]. In particular, ACC pathways innervate mostly excitatory pyramidal neurons across OFC layers, which in turn project back to all ACC layers [8]. Interactions between ACC and OFC have distinct implications for psychiatric diseases, such as obsessive–compulsive disorder and phobias [80–82]. It would be expected from our results that reduction of both excitatory neurons and myelinated axons in OFC would disrupt connectivity and communication with ACC and other medial prefrontal cortices. In line with this, previous studies in the white matter showed disrupted axon morphology under both ACC and OFC in brains from individuals with ASD, indicating compromised communication [27, 37].

Finally, the OFC has among the most robust reciprocal connections with the amygdala [10–12, 43, 83–86], forming a key circuit for affective processing. Anatomically, amygdala projections to OFC follow a feedback pattern, originating mostly from the basolateral nucleus that mainly innervates excitatory pyramidal, as well as CB and CR inhibitory neurons in the upper layers of OFC, modulating local activity [11]. OFC neurons in middle and deep layers (mostly 3 and 5) project back to the amygdala, targeting robustly and preferentially inhibitory neurons in the intercalated masses (IM), in pathways that regulate autonomic function and play a key role in learning affective associations [10, 87]. Reduction of top-down OFC control of the inhibitory output from IM may lead to excessive autonomous or impulsive behaviors. In line with this, lesions in the OFC or amygdala frequently result in emotional processing deficits seen typically in ASD, including carelessness and lack of affect [88, 89]. Previous studies also showed that in ASD there is an initial excess of neurons in the amygdala during childhood, followed by a reduction in the density of neurons in adulthood across nuclei [90, 91]. The decreased density of OFC neurons and axons in adults with ASD observed in our study provides evidence for parallel development of pathology in amygdala and OFC in ASD. This is also supported by functional studies that showed reduced amygdala-OFC activity in adults with ASD [68], and structural studies that showed reduced tract volume and lower mean fractional anisotropy values in the uncinate fasciculus, the axon bundle connecting OFC and the temporal lobe, including the amygdala [28, 29, 92].

## Limitations

Availability of post-mortem brain tissue that is optimally preserved for correlated quantitative light and electron microscopy studies and immunohistochemical staining was a limiting factor that determined the sample size of this study. To minimize the effects of limited tissue availability we minimized variability in age and PMI, using only right hemispheres from adults with similar PMI. Our subjects’ ages were restricted between 30–67 years, a relatively narrow range, with minimal variability in the density of neurons and myelinated axons in the prefrontal cortex [27, 36, 37]. As such, our final sample size was adequate for this age range and similar to other published studies using comparable approaches. Using this approach, we observed laminar distribution and density patterns of neurons and axons in the neurotypical OFC that were in line with previous findings in the cortex of primates [27, 36, 37, 42, 93–100]. Importantly, we found robust differences between Control and ASD groups, suggesting that differences between the two groups would remain highly significant with the addition of more subjects in future studies.

## Conclusions

The balance between excitation and inhibition in OFC networks is at the core of their functionality, in assessing and integrating emotional and social cues with internal states and external inputs. Our preliminary results provide evidence for laminar-specific changes in the ratio of excitation/inhibition in OFC of adults with ASD, with an overall weakening and disorganization of excitatory inputs and outputs, and a relative strengthening of local inhibition. These changes likely have widespread effects on major OFC communications with neighboring limbic or distant sensory association cortices, and the top-down control of the amygdala in individuals with ASD, and provide the anatomic basis for disrupted transmission of signals for social interactions and emotions in autism.

## Data Availability

The datasets used and/or analyzed during the current study are available from the corresponding author on reasonable request.

## Abbreviations

ACC: Anterior cingulate cortices
ASD: Autism Spectrum Disorders
EM: Electron microscopy
IM: Intercalated masses
MPFC: Medial prefrontal cortices
OFC: Orbitofrontal cortices
PMI: Post-mortem interval
